# Location, sex, and age disparities in trend of epidemiology of anxiety disorder in the Middle East and North Africa: Potential effects of COVID-19 on estimates

**DOI:** 10.1097/MD.0000000000049665

**Published:** 2026-07-10

**Authors:** Moien AB Khan, Sohrab Amiri, Abolghasem Yaghoobi, Fatemehjan Nikbakht Fini

**Affiliations:** a Department of Family Medicine, Health and Wellness Research Group, College of Medicine and Health Sciences, United Arab Emirates University, Al-Ain, United Arab Emirates; b Spiritual Health Research Center, Lifestyle Institute, Baqiyatallah University of Medical Sciences, Tehran, Iran; c Department of Psychology, Faculty of Economics and Social Sciences, Bu-Ali Sina University, Hamedan, Iran; d Department of General Psychology, Faculty of Literature, Humanities and Social Sciences, Science and Research Branch, Islamic Azad University, Tehran, Iran.

**Keywords:** anxiety disorder, Global Burden of Disease, incidence, Middle East and North Africa, prevalence

## Abstract

This study aims to provide an epidemiological estimate of the burden of anxiety disorders based on gender and age disparity in 21 Middle East and North African (MENA) countries and also present potential effects of COVID-19 on estimates. The tools utilized for this research included Global Burden of Disease compare and result from the Global Burden of Disease Study. All-age count estimates and an age-standardized rate (per 100,000) were calculated for prevalence, incidence, and disability-adjusted life years. Each of the disease burden indicators was examined from 1990 to 2021, stratified by sex, age, and location, and the percentage change between 1990 and 2021 was reported. The 95% uncertainty interval was reported for each of the reported estimates. Increase in anxiety disorders during 2020 and 2021, used to estimate likely connected to the impacts of COVID-19. The global prevalence of anxiety disorders has increased from 3746 per 100,000 people to 4421 per 100,000 people. The same estimate in the MENA was equal to 4945 per 100,000 in 1990 and 5950 per 100,000 in 2021. In 2021, there were 359 million cases of anxiety disorders globally, of which 38 million were in the MENA. There was an increase in disability-adjusted life years because of anxiety disorder during 2020 and 2021, coinciding with the pandemic period. The trend of burden of anxiety disorders in the global and MENA countries has shown an increasing trend during the recent decades that has continued since 1990. However, the rate of anxiety disorders in the MENA was higher than globally. The COVID-19 pandemic may have significantly contributed to a rise in anxiety disorders, potentially placing a long-term strain on the mental health system for years to come.

## 1. Introduction

Anxiety disorders are among the most common mental disorders that usually begin before adulthood.^[1]^ These disorders are biopsychosocial conditions that are associated with responses to general or specific threats.^[2]^ The prevalence of anxiety disorders has been investigated and has attracted significant attention.^[3,4]^ The report of the World Health Organization states that 301 million cases of anxiety disorder were estimated in the world in 2019, in this report, women are known to be more affected by anxiety disorders compared with men.^[5]^ New cases of anxiety disorder account for 45.8 million people in the world.^[6]^ Although the age-adjusted pattern of anxiety disorder in the world has remained almost constant during the last 3 decades, the absolute number of anxiety cases has increased by 50%.^[6]^ Anxiety disorders are increasing in incidence worldwide, but there are many geographical disparities in anxiety disorders that complicate interventions.^[6]^

The epidemiology of a disorder is critical to understanding whether anxiety disorders are on the rise, understanding population trends, factors associated with the disorder, and factors that can be effective in understanding and controlling anxiety disorders.^[7]^ Globally, there is a disparity in the prevalence of anxiety disorders based on geographic diversity, age, gender, and other factors.^[6]^ The Middle East and North Africa (MENA) are regions in the world where the health systems of countries in this region have been associated with challenges in growth and improvements,^[8]^ and the prevalence of mental disorders, especially anxiety, has had a significant burden, and this disparity in the prevalence of mental disorders in this region has been historically noted.^[9,10]^ The last published report on the epidemiology of mental disorders in MENA belongs to the Global Burden of Disease (GBD) 2019; its findings show a 13.8% increase in disability-adjusted life-years (DALY) caused by mental disorders between 1990 and 2019.^[10]^ The age-standardized prevalence rate of anxiety disorders was 5135 per 100,000 in 2019 with a percentage change (%) of 3.74.^[10]^

Over the past decades, health systems in MENA have changed, as have demographic changes and population growth, which have increased the need for access to mental health care.^[11,12]^ In recent decades, this region has witnessed conflicts, wars, migrations, and displacements, all of which have affected various dimensions of health, especially anxiety disorders.^[13]^ On the other hand, COVID-19 had a significant impact on the prevalence of mental disorders.^[14,15]^ However, previous estimates of the burden of anxiety disorders in MENA based on estimates of the GBD did not cover the effects of COVID-19.^[10]^

This study aims to provide an epidemiological estimate of the burden of anxiety disorder based on gender and age disparity in 21 MENA countries. Also, in this study, the increase in anxiety disorders during 2020 and 2021 was used to estimate likely connections to the impacts of COVID-19. Also, the global burden of anxiety disorder was presented.

## 2. Methods

### 2.1. Data source

The present study reports descriptive statistics extracted from GBD outputs with no independent modeling beyond data retrieval. This study analyzed data from the GBD 2021, focusing on the MENA super region, which comprises 21 countries. The GBD 2021 dataset offers extensive information on prevalence, incidence, DALYs, years lived with disability (YLDs), years of life lost (YLLs), mortality associated with 371 diseases and injuries, and healthy life expectancy. These annual estimates span the years from 1990 to 2021, covering 7 super regions and 204 countries, with data categorized by age, sex, Sociodemographic Index, and subnational levels for select countries.^[16]^ The GBD 2021 utilized a total of 100,983 data sources, which included 19,189 new sources specifically for DALYs. In addition, it introduced 12 new causes and implemented notable methodological improvements. Further details about the methodology and outcomes of the GBD can be found in associated research publications.^[16]^ This data extraction was conducted utilizing tools from the GBD study, specifically GBD Results and GBD Compare. No independent modeling or statistical analyses were conducted beyond the retrieval of data from the GBD study. This data extraction was done in 2025.

### 2.2. Case definitions

In the GBD,^[16]^ anxiety disorders are characterized by periods of extreme fear and distress, frequently paired with a range of physical symptoms. The aim of our study was to encompass all cases of anxiety disorders that align with the diagnostic standards outlined in the Diagnostic and Statistical Manual of Mental Disorders (DSM) or the International Classification of Diseases (ICD) as defined by the World Health Organization.^[17,18]^ The anxiety disorders considered encompassed panic disorder, agoraphobia, specific phobias, social phobia, obsessive-compulsive disorder, post-traumatic stress disorder, generalized anxiety disorder – which includes overanxious disorder in childhood – separation anxiety disorder, and anxiety disorders classified as “not otherwise specified.” The following codes were used for identification: DSM-IV-Text Revision (TR): 300.0 to 300.3, 208.3, 309.21, 309.81; ICD-10: F40 to F42, F43.0, F43.1, F93.0 to F93.2, F93.8. Anxiety disorders caused by medical conditions or substances were excluded. Various versions of DSM (III, III-R, IV, IV-TR, 5, 5-TR) and ICD (9, 10, 11) were included.^[17,18]^ Anxiety disorders were collectively addressed as a single category to avoid duplicating the counts of individuals who fulfilled the diagnostic criteria for multiple anxiety disorders. Epidemiological data reporting outcomes for combined or overall anxiety disorders were included in the analyses, provided they encompassed at least 3 distinct types of anxiety disorders.^[16]^

### 2.3. Estimation framework

YLDs were calculated through a microsimulation process that utilized estimated prevalence rates of nonfatal disease outcomes, specific to age, sex, location, and year. These prevalence estimates were matched with corresponding disability weights for each outcome, adjusted to account for different severity levels using appropriate scaling. YLLs were determined by multiplying the estimated number of deaths – categorized by age, sex, location, and year – with the standard life expectancy at the age of death for a given cause. Ultimately, DALYs were obtained by combining YLDs and YLLs.^[16]^

### 2.4. Statistical analysis

All-age count estimates and age-standardized rates (per 100,000) were extracted directly from GBD outputs for prevalence, incidence, and DALYs. Each of the disease burden indicators was examined in the period of 1990 to 2021, stratified by sex, age, and location, and the % change between 1990 and 2021 was reported. The 95% uncertainty interval was reported for each of the reported estimates. More details about data, data processing, and modeling are available elsewhere, related to GBD 2021^[16]^. The GBD does not specifically detail the exact methodology used to assess the effects of COVID-19. At most, the authors acknowledge observing a rise in anxiety disorders during 2020 and 2021, which is likely linked to COVID-19. The manuscript utilizes descriptive statistics and leverages GBD’s integrated Bayesian meta-regression framework to estimate uncertainty. GBD 2021 complies with the Guidelines for Accurate and Transparent Health Estimates Reporting.^[19]^

### 2.5. Ethics approval

Ethics approval was not required for analysis of publicly available, de-identified data.

## 3. Results

### 3.1. Prevalence of anxiety disorders in MENA from 1990 to 2021

**Figure 1.**
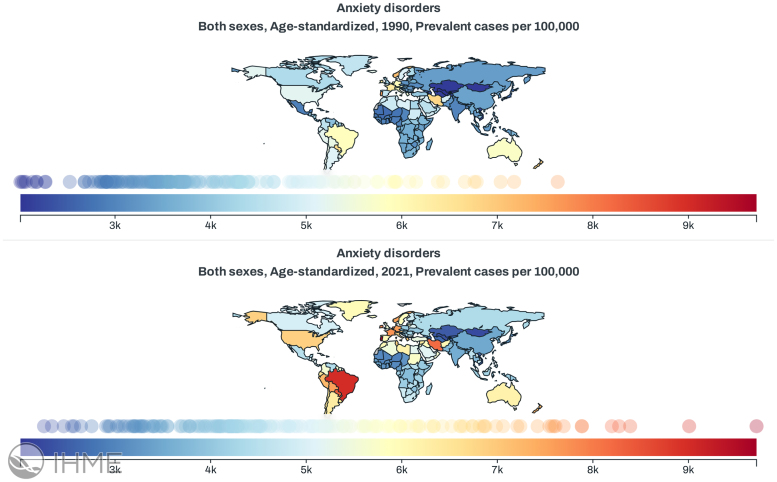
The global age-standardized prevalence rate of anxiety disorders, 1990 to 2021.

**Figure 2.**
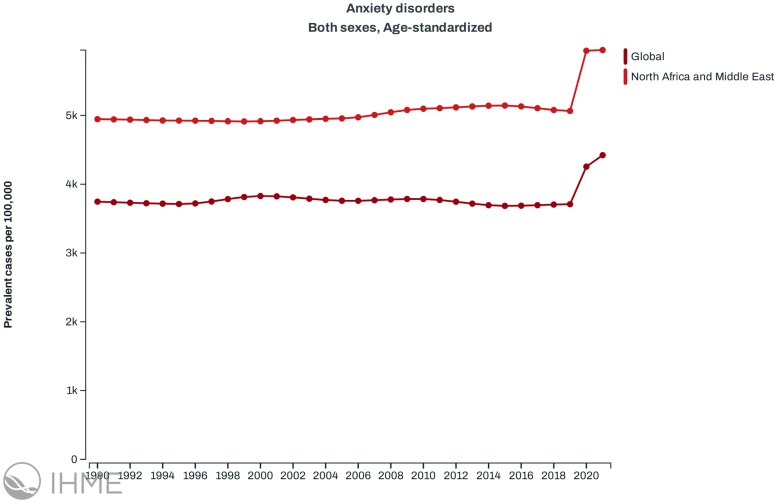
The trend of age-standardized prevalence rate of anxiety disorders, global and MENA. MENA = Middle East and North Africa.

The age-standardized prevalence rate of anxiety disorders showed an increase in the prevalence of anxiety disorders from 1990 to 2021 both globally and in the MENA super region. The global prevalence of anxiety disorders has increased from 3746 per 100,000 people to 4421 per 100,000 people. The same estimate in MENA was 4945 per 100,000 in 1990 and 5950 per 100,000 in 2021. This shows that the prevalence of anxiety disorders in MENA has increased faster than globally. In 2021, there were 359 million cases of anxiety disorders globally, of which 38 million were in MENA (Table 1, Figs. 1 and 2).

**Table 1:** All-ages counts and age-standardized prevalence, incidence, DALYs rates (per 100,000) of anxiety disorder in the Middle East and North Africa, 1990 to 2021.

Location	Year					
	1990			2021		
	Value	Lower	Upper	Value	Lower	Upper
Age-standardized prevalence rate (per 100,000)						
Global	3746.36	3234.69	4368.51	4421.87	3768.28	5182.08
MENA	4945.43	4195.92	5812.07	5950.26	4892.21	7231.97
Age-standardized incidence rate (per 100,000)						
Global	562.48	468.32	686.07	678.25	565.15	832.44
MENA	728.07	606.87	902.13	883.41	722.42	1108.54
Age-standardized DALYs rate (per 100,000)						
Global	443.73	306.21	603.28	524.33	363.06	716.25
MENA	587.75	407.24	806.3	707.08	469.77	994.9
Prevalence counts estimates						
Global	192,936,133.81	164,639,913.66	226,523,808.92	359,213,270.22	307,185,425.26	419,966,576.52
MENA	15,881,329.48	13,275,923.77	19,086,607.03	37,799,731.40	30,917,632.50	46,289,420.78
Incidence counts estimates						
Global	30,239,332.56	25,024,016.67	37,400,423.54	53,917,371.01	44,990,957.62	65,978,474.77
MENA	2,583,166.97	2,106,820.55	3,173,584.03	5,702,847.41	4,644,051.75	7,167,442.13
DALYs counts estimates						
Global	22,996,731.91	15,814,115.76	31,547,275.96	42,509,645.28	29,396,722.99	57,729,820.42
MENA	1,908,924.92	1,313,103.31	2,684,061.16	4,508,067.43	2,969,187.28	6,368,320.64

DALYs = disability-adjusted life-years, MENA = Middle East and North Africa.

### 3.2. Incidence of anxiety disorders in MENA from 1990 to 2021

**Figure 3.**
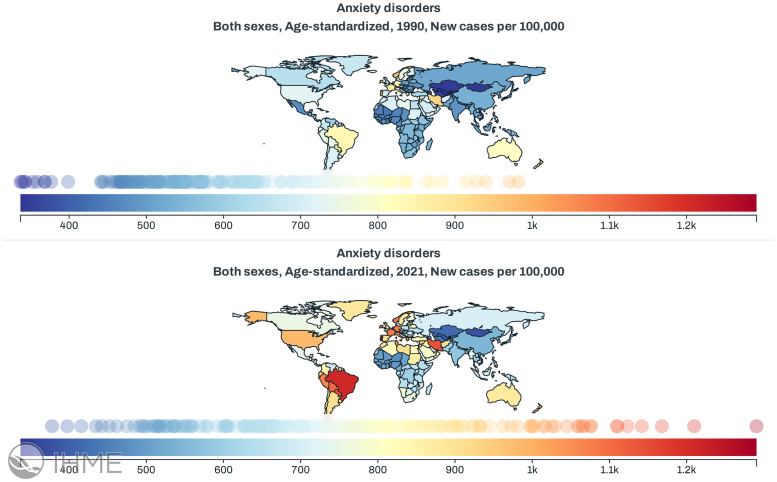
The global age-standardized incidence rate of anxiety disorders, 1990 to 2021.

The new cases of anxiety disorders in 2021 globally and in MENA have increased compared with the previous decade, that is, 1990, and this increase was greater in MENA. The global age-standardized incidence rate was 678 per 100,000, equivalent to 54 million incidences of anxiety disorder. This rate in MENA was 883 per 100,000, equivalent to 5.7 million incidences of anxiety disorder. In 2021, the MENA region accounted for approximately 5.7 million incidence cases out of a global total of 53.9 million, representing nearly 10% of the worldwide figure (Table 1, Fig. 3).

### 3.3. DALYs of anxiety disorders in MENA from 1990 to 2021

**Figure 4.**
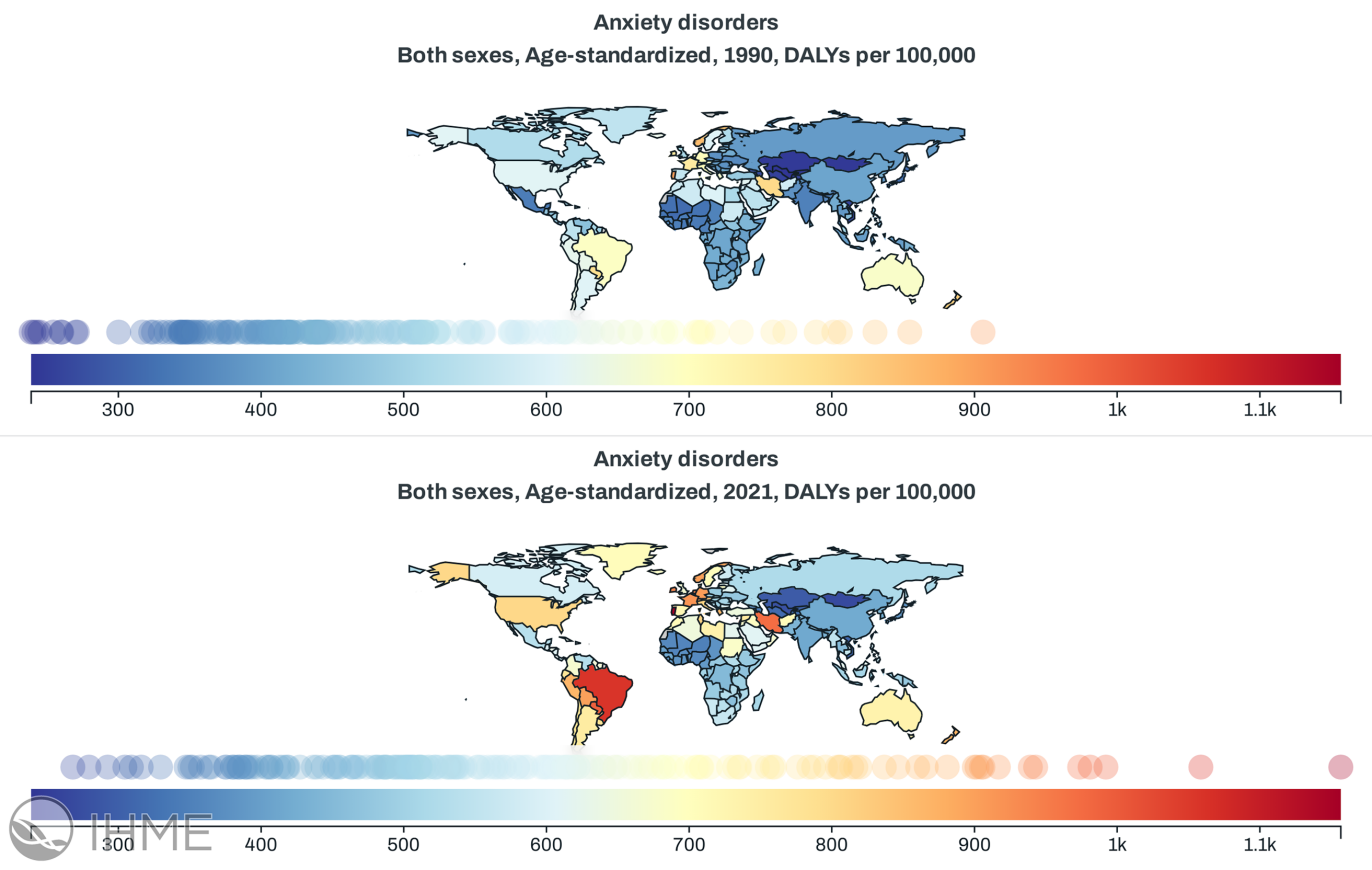
Global age-standardized DALYs rate of anxiety disorders, 1990 to 2021. DALYs = disability-adjusted life-years.

**Figure 5.**
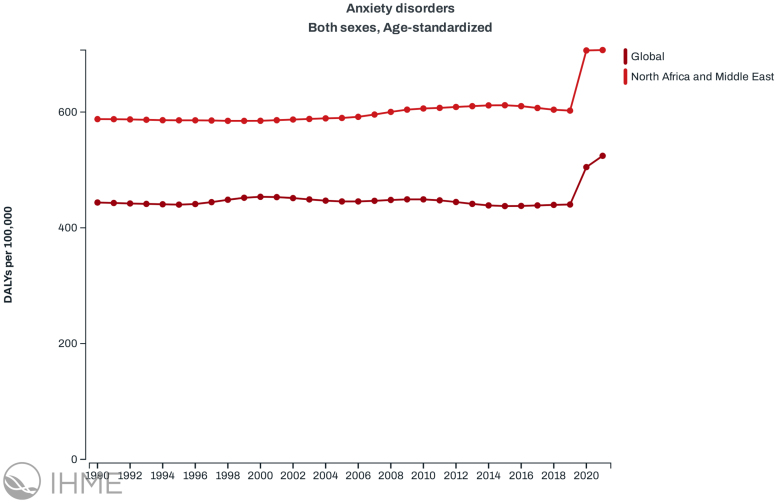
The trend of age-standardized DALYs rate of anxiety disorders, global and MENA. DALYs = disability-adjusted life-years, MENA = Middle East and North Africa.

Global age-standardized DALY rates increased from 1990 to 2021 to 524 per 100,000 people, which was equal to 42.5 million DALYs. This rate was 707 per 100,000 for MENA, which was equal to 4.5 million DALYs. DALYs count estimates because of anxiety disorders in 2021 more than doubled compared with 1990 (Table 1, Fig. 4). In 2019, coinciding with the onset of the COVID-19 pandemic, there was a notable and significant escalation in DALYs attributed to anxiety disorders. This marked surge reflects the widespread psychological and emotional challenges that emerged during this unprecedented global crisis, highlighting the profound impact of the pandemic on mental health and overall societal well-being (Fig. 5).

### 3.4. The sex-specific burden of anxiety disorders in MENA, 2021

There was a disparity in the burden of anxiety disorders by gender. The age-standardized prevalence rate (per 100,000) of anxiety disorders in males was 4452, and in females, it was 7566. This gender disparity in the prevalence of anxiety disorders has existed since 1990. Out of a total of 38 million cases of anxiety disorders in 2021, 14.7 million were males and 23 million were females. Anxiety disorders caused 1.8 million DALYs in males and 2.7 million DALYs in females. New cases of anxiety disorders also show that gender disparity persisted from 1990 to 2021 and the incidence of anxiety disorders is higher in females (Table 2).

**Table 2:** All-ages counts and age-standardized prevalence, incidence, DALYs rates (per 100,000) of anxiety disorder in the Middle East and North Africa, stratified by sex, 1990 to 2021.

Sex	Year					
	1990			2021		
	Value	Lower	Upper	Value	Lower	Upper
Age-standardized prevalence rate (per 100,000)						
Males	3720.35	3107.02	4369.25	4452.54	3641.55	5440.51
Females	6228.31	5240.02	7420.57	7566.04	6174.88	9273.69
Age-standardized incidence rate (per 100,000)						
Males	644.26	535.14	769.69	778	637.36	960.09
Females	816.16	674.19	1023.43	996.95	799.36	1286.09
Age-standardized DALYs rate (per 100,000)						
Males	448.32	308.15	619.22	537.11	358.5	754.75
Females	733.8	500.32	997.39	890.44	594.36	1245.14
Prevalence counts estimates						
Males	6,205,130.27	5,159,488.56	7,514,688.01	14,735,050.61	11,926,051.13	18,073,129.14
Females	9,676,199.20	8,050,232.38	11,689,423.78	23,064,680.80	18,678,188.19	28,401,344.39
Incidence counts estimates						
Males	1,160,565.57	952,181.50	1,410,119.26	2,603,541.44	2,105,771.15	3,235,897.72
Females	1,422,601.40	1,148,248.37	1,802,356.18	3,099,305.97	2,469,682.17	4,019,908.93
DALYs counts estimates						
Males	755,088.39	514,097.38	1,075,188.68	1,784,165.88	1,171,630.95	2,513,690.38
Females	1,153,836.53	784,655.10	1,597,904.34	2,723,901.55	1,807,191.68	3,838,236.08

DALYs = disability-adjusted life-years.

### 3.5. The age-specific burden of anxiety disorders in MENA, 2021

Age disparity in the burden of anxiety disorders was shown. There was a higher prevalence of anxiety disorders in younger age groups. The highest prevalence of anxiety disorders was at the age of 10 to 19 years with 9 million cases of anxiety disorders. At the same age, there were 1.5 million new cases of anxiety disorders, with 1114,878 DALYs count estimates (Table 3).

**Table 3:** All-ages counts and prevalence, incidence, DALYs rates (per 100,000) of anxiety disorder in the Middle East and North Africa, stratified by age, 1990 to 2021.

Age (yr)	Year					
	1990			2021		
	Value	Lower	Upper	Value	Lower	Upper
Prevalence rate (per 100,000)						
5–9	2344.97	1610.52	3310.27	3002.89	1969.93	4426.21
10–19	6484.43	4743.94	8574.10	8072.54	5684.14	11,228.37
20–24	6853.54	5085.92	9133.59	8367.44	5888.13	11,405.03
25–29	6459.03	4768.36	8742.49	7889.88	5520.02	10,949.12
30–34	6163.22	4724.26	8074.17	7557.98	5556.94	10,308.63
35–39	5933.05	4601.20	7595.28	7206.79	5367.82	9386.72
40–44	5729.66	4170.92	7393.11	6831.63	4805.39	9200.75
45–49	5517.75	4000.86	7267.32	6457.17	4446.82	8665.34
50–69	5050.89	3991.96	6521.63	5732.66	4419.19	7548.58
70+	4159.50	3123.13	5649.88	4534.57	3293.10	6169.78
Prevalence counts estimates						
5–9	1,113,648.88	764,850.17	1,572,079.31	1,897,879.75	1,245,029.75	2,797,437.63
10–19	5,061,200.43	3,702,724.48	6,692,226.24	9,053,582.17	6,374,929.74	12,592,934.92
20–24	2,114,622.83	1,569,232.25	2,818,119.38	4,195,785.49	2,952,555.94	5,718,963.11
25–29	1,696,660.81	1,252,553.46	2,296,480.22	3,903,161.75	2,730,781.75	5,416,585.03
30–34	1,359,753.42	1,042,285.12	1,781,356.74	3,847,241.69	2,828,655.42	5,247,411.48
35–39	1,089,178.95	844,680.20	1,394,327.01	3,645,105.50	2,714,975.39	4,747,685.47
40–44	838,017.82	610,038.07	1,081,313.93	2,996,654.46	2,107,854.27	4,035,852.76
45–49	652,036.88	472,784.24	858,784.25	2,337,535.91	1,609,776.29	3,136,908.15
50–69	1,576,496.18	1,245,980.25	2,035,545.99	4,873,493.41	3,756,878.05	6,417,254.14
70+	300,384.29	225,541.25	408,014.57	922,061.64	669,620.77	1,254,566.28
Incidence rate (per 100,000)						
5–9	975.7	674.27	1389.95	1252.16	833.5	1822.64
10–19	1101.38	852.87	1426.84	1382.71	1018.88	1846.02
20–24	824.19	438.35	1265.88	1018.37	513.81	1586.32
25–29	812.64	575.59	1132.28	989.3	677.03	1400.64
30–34	801.43	579.89	1076.25	962.68	682.8	1307.71
35–39	789.75	525.4	1112.00	928.66	599.84	1344.05
40–44	752.93	490.74	1075.16	868.51	542.06	1278.58
45–49	691.53	495.55	940.36	792.19	547.63	1101.49
50–69	558.42	394.07	789.36	629.87	435.29	876.82
70+	303.45	223.07	409.99	321.2	231.31	427.83
Incidence counts estimates						
5–9	463,369.56	320,216.18	660,101.85	791,388.25	526,786.51	1151,937.14
10–19	859,642.95	665,677.17	1113,674.56	1550,749.56	1142,706.61	2070,368.12
20–24	254,298.01	135,249.71	390,581.55	510,652.18	257,643.84	795,449.87
25–29	213,465.20	151,195.37	297,426.64	489,413.81	334,929.03	692,902.81
30–34	176,813.90	127,937.30	237,447.56	490,032.15	347,567.08	665,662.82
35–39	144,979.97	96,451.13	204,138.95	469,706.81	303,392.15	679,801.84
40–44	110,123.21	71,775.10	157,252.84	380,967.91	237,771.07	560,840.79
45–49	81,718.13	58,559.39	111,122.70	286,778.64	198,246.00	398,746.19
50–69	174,296.43	122,996.49	246,377.22	535,472.39	370,053.72	745,407.78
70+	21,913.89	16,109.41	29,608.02	65,312.67	47,035.30	86,994.59
DALYs rate (per 100,000)						
5–9	292.97	173.75	444.22	376.31	214.44	604.74
10–19	797.69	494.03	1174.91	994.07	605.57	1521.57
20–24	830.85	523.14	1248.66	1014.50	608.62	1552.00
25–29	774.06	476.81	1157.40	946.12	567.62	1444.15
30–34	733.12	475.19	1067.07	899.61	562.37	1324.75
35–39	701.14	462.46	999.9	851.42	535.48	1231.17
40–44	672.63	413.22	996.44	800.57	476.11	1187.36
45–49	643.87	393.43	957.8	750.87	442.85	1122.53
50–69	578.36	389.02	831.17	650.92	418.09	955.87
70+	445.97	294.41	653.76	480.49	306.67	705.22
DALYs counts estimates						
5–9	139,133.03	82,514.00	210,964.11	237,835.08	135,528.42	382,205.53
10–19	622,612.78	385,596.11	917,036.74	1,114,878.63	679,166.93	1,706,487.28
20–24	256,354.09	161,410.40	385,265.62	508,715.56	305,190.15	778,238.66
25–29	203,329.83	125,249.59	304,027.54	468,049.74	280,802.97	714,426.12
30–34	161,743.17	104,838.31	235,421.58	457,928.04	286,263.88	674,340.83
35–39	128,713.74	84,896.84	183,560.42	430,636.84	270,841.11	622,710.88
40–44	98,378.50	60,438.02	145,738.78	351,167.29	208,843.01	520,826.43
45–49	76,086.18	46,491.52	113,184.17	271,818.15	160,313.54	406,362.74
50–69	180,519.95	121,422.01	259,427.62	553,362.18	355,429.09	812,613.79
70+	32,206.07	21,261.30	47,212.47	97,703.38	62,357.97	143,399.60

DALYs = disability-adjusted life-years.

### 3.6. Burden of anxiety disorder at the national level

**Figure 6.**
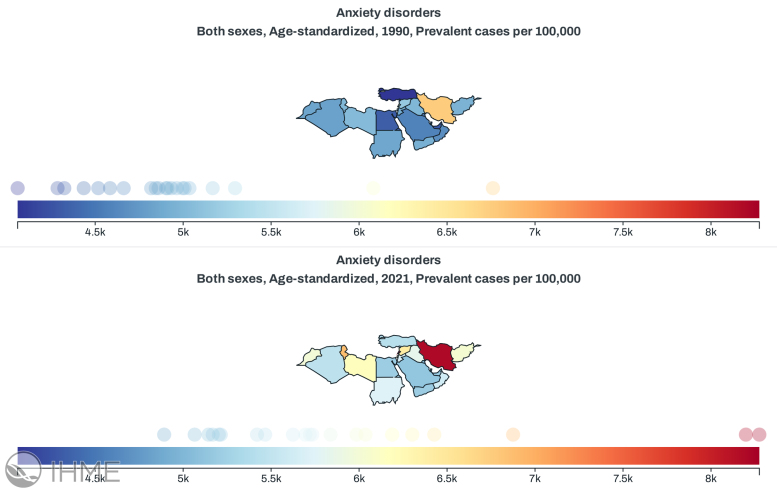
The age-standardized prevalence rate of anxiety disorders in MENA, stratified by country, 1990 to 2021. MENA = Middle East and North Africa.

**Figure 7.**
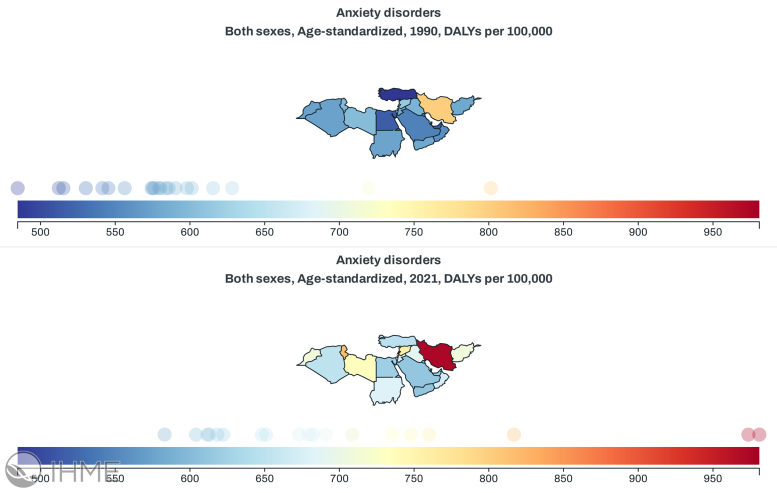
Age-standardized DALYs rate of anxiety disorders in MENA, stratified by country, 1990 to 2021. DALYs = disability-adjusted life-years, MENA = Middle East and North Africa.

There was a disparity in the burden of anxiety disorders in MENA countries. In 2021, Lebanon had the highest prevalence of anxiety disorders. The age-standardized prevalence rate for anxiety disorders was 8274 (95% uncertainty interval = 5766–11,065) per 100,000 in Lebanon. This included 473,053 cases of anxiety disorders. The lowest prevalence was in Qatar. Iran had the highest number of anxiety disorders with 7.3 million cases. After Iran, Egypt and Türkiye followed (Table 4, Fig. 6). The highest number of new cases of anxiety disorders in Lebanon was 1168 per 100,000 people. Iran and Tunisia ranked next. The lowest rate of anxiety disorders was in Kuwait (Table 5). The highest DALYs were caused by anxiety disorders in Lebanon and then in Iran. The highest counts of DALYs because of anxiety disorders were in Iran with 864,198 (95% uncertainty interval = 587,427–1191,909; Table 6, Fig. 7).

**Table 4:** All-ages counts and age-standardized prevalence rates (per 100,000) of anxiety disorder in the Middle East and North Africa, stratified by country, 1990 to 2021.

Location	Year					
	1990			2021		
	Value	Lower	Upper	Value	Lower	Upper
Age-standardized prevalence rate (per 100,000)						
MENA	4945.43	4195.92	5812.07	5950.26	4892.21	7231.97
Afghanistan	4963.84	4024.73	6114.80	6036.08	4354.20	8133.31
Algeria	4818.36	3896.04	5978.73	5466.45	3865.66	7367.72
Bahrain	4911.09	3964.23	6043.29	5719.99	4016.02	7508.78
Egypt	4324.78	3470.06	5364.35	5213.93	3730.07	7105.72
Iran	6760.71	5887.28	7737.18	8198.38	7057.54	9426.24
Iraq	4996.83	4026.34	6145.86	5837.95	4136.59	7886.99
Jordan	4902.54	3986.02	6058.81	5694.68	4016.10	7541.96
Kuwait	4517.75	3656.17	5571.08	5065.57	3632.52	6817.41
Lebanon	6081.11	4914.00	7498.07	8274.66	5766.56	11,065.29
Libya	5035.79	4089.83	6125.25	6188.30	4447.95	8519.96
Morocco	4844.47	3919.73	5942.05	5985.12	4316.49	8092.99
Oman	4662.31	3772.07	5691.81	5624.34	3947.31	7639.36
Palestine	5295.00	4256.02	6493.62	6302.59	4552.02	8392.60
Qatar	4435.23	3583.85	5402.29	4892.21	3499.36	6708.93
Saudi Arabia	4584.65	3698.45	5704.74	5144.07	3714.57	6929.05
Sudan	4860.66	3938.58	6023.39	5734.42	4172.57	8075.87
Syrian Arab Republic	5168.04	4158.22	6416.84	6427.35	4549.33	8864.77
Tunisia	5008.36	4055.92	6110.62	6875.36	4987.43	9496.72
Türkiye	4059.68	3683.80	4492.10	5420.06	3786.57	7370.38
United Arab Emirates	4286.51	3534.25	5169.82	5168.10	3648.71	7062.59
Yemen	4933.60	3979.32	6133.26	5202.99	3785.27	6971.28
Prevalence counts estimates						
MENA	15,881,329.48	13,275,923.77	19,086,607.03	37,799,731.40	30,917,632.50	46,289,420.78
Afghanistan	446,420.22	358,286.66	558,908.98	1780,311.39	1256,459.87	2,503,673.29
Algeria	1,154,354.45	906,540.68	1,474,720.20	2,405,796.56	1,696,837.82	3,263,058.66
Bahrain	24,881.96	19,518.79	31,868.99	93,622.32	66,062.62	123,860.10
Egypt	2,281,368.07	1,796,766.77	2,855,480.91	5,481,791.02	3,911,372.48	7,472,413.55
Iran	3,523,365.86	3,025,159.12	4,099,969.92	7,288,282.29	6,217,243.91	8,378,424.63
Iraq	846,004.31	663,620.33	1,067,692.45	2,460,205.71	1,733,540.74	3,379,014.48
Jordan	173,038.24	135,205.59	221,038.36	737,435.55	513,268.09	978,697.36
Kuwait	78,852.76	62,011.84	100,188.21	256,067.81	181,744.14	346,023.53
Lebanon	176,425.10	140,419.55	219,239.14	473,053.08	331,020.07	634,185.69
Libya	202,542.15	159,393.40	257,900.37	458,304.15	328,435.53	626,544.53
Morocco	1,187,841.96	937,984.70	1,484,508.03	2,282,347.91	1,643,839.19	3,088,909.80
Oman	85,673.62	67,624.25	107,668.84	271,739.86	189,928.20	370,977.14
Palestine	96,291.04	75,661.72	122,697.45	327,023.50	230,094.36	445,128.29
Qatar	20,010.90	15,712.14	25,555.81	155,077.72	107,587.60	219,878.87
Saudi Arabia	695,906.42	540,235.04	885,947.67	2,108,231.05	1,495,959.13	2,885,792.38
Sudan	894,634.11	706,553.64	1,135,002.81	2,497,204.91	1,789,131.38	3,544,144.28
Syrian Arab Republic	602,714.11	472,649.89	766,792.18	945,250.94	668,282.92	1,288,268.14
Tunisia	409,818.00	324,255.66	513,669.77	826,675.76	598,155.39	1,122,049.28
Türkiye	2316,873.09	2,079,163.46	2,560,139.59	4,694,266.17	3,283,842.85	6,411,242.95
United Arab Emirates	79,358.63	64,650.48	98,270.80	519,113.71	358,344.80	711,671.06
Yemen	576,266.72	451,779.55	736,561.00	1,702,674.01	1,218,061.09	2,315,535.88

DALYs = disability-adjusted life-years, MENA = Middle East and North Africa.

**Table 5:** All-ages counts and age-standardized incidence rates (per 100,000) of anxiety disorder in the Middle East and North Africa, stratified by country, 1990 to 2021.

Location	Year					
	1990			2021		
	Value	Lower	Upper	Value	Lower	Upper
Age-standardized incidence rate (per 100,000)						
MENA	728.07	606.87	902.13	883.41	722.42	1108.54
Afghanistan	722.27	589.62	916.93	901.41	640.42	1264.13
Algeria	710.08	583.08	897.17	812.81	572.86	1135.81
Bahrain	728.87	592.2	916.13	884.63	622.2	1191.85
Egypt	660.51	536.59	828.44	813.5	581.6	1118.19
Iran	927.98	765.82	1132.52	1142.02	938.58	1401.35
Iraq	718.33	588.63	904.55	846.84	608.51	1170.40
Jordan	720.81	586.47	912.63	852.48	609.34	1182.44
Kuwait	691.13	564.5	865.62	761.38	556.43	1055.96
Lebanon	820.21	670.82	1034.73	1168.05	824.02	1570.70
Libya	735.89	603.61	938.88	910.93	645.24	1275.26
Morocco	713	581.69	905.25	894.56	658.93	1198.88
Oman	705.5	570.67	892.04	869.88	624.08	1158.12
Palestine	750.61	602.69	939.58	921.22	657.65	1246.88
Qatar	691.2	568.99	863.68	773.42	547.49	1069.20
Saudi Arabia	695.99	564.05	876.77	788.51	572.31	1076.11
Sudan	714.03	581.64	910.93	858.75	612.38	1211.25
Syrian Arab Republic	746.62	604.56	951.27	923.99	659.93	1258.74
Tunisia	728.53	593.83	927.84	1025.71	722.51	1475.05
Türkiye	630.69	530.38	738	841.34	608.36	1151.42
United Arab Emirates	675.59	555.32	850.33	835.42	606.53	1165.59
Yemen	720.73	584.62	914.42	769.08	553.55	1044.65
Incidence counts estimates						
MENA	2,583,166.97	2,106,820.55	3,173,584.03	5,702,847.41	4,644,051.75	7,167,442.13
Afghanistan	71,863.07	57,817.32	90,890.46	303,477.38	212,741.85	426,471.61
Algeria	189,431.17	152,648.54	239,455.84	363,902.59	255,477.84	506,148.07
Bahrain	3873.85	3109.17	5029.62	13,860.60	9612.59	18,860.02
Egypt	377,618.60	302,045.10	471,559.46	899,196.81	636,150.25	1,243,266.09
Iran	558,533.75	459,403.06	686,611.79	986,388.52	808,524.84	1,216,358.92
Iraq	137,998.47	110,289.71	175,267.27	373,399.29	263,928.03	520,561.46
Jordan	28,551.75	22,684.13	36,609.49	112,466.18	80,016.40	156,130.00
Kuwait	12,545.14	10,100.97	15,951.65	36,402.73	26,141.37	50,368.20
Lebanon	25,081.81	20,504.19	31,627.26	63,879.34	45,411.33	85,516.46
Libya	33,051.80	26,353.42	41,514.33	65,019.37	45,650.18	89,924.07
Morocco	188,244.15	151,841.33	242,203.79	336,701.42	246,908.99	452,167.98
Oman	14,539.96	11,637.48	18,442.88	42,559.30	30,712.85	57,926.06
Palestine	15,790.36	12,500.91	19,956.81	51,315.22	36,396.60	70,568.57
Qatar	3186.30	2531.40	4075.35	23,538.54	15,858.60	32,553.29
Saudi Arabia	116,422.77	92,019.84	146,045.88	310,803.07	223,059.92	432,304.83
Sudan	147,189.37	116,506.90	187,214.06	403,700.60	280,069.88	577,616.56
Syrian Arab Republic	100,131.95	79,515.93	126,036.62	134,787.91	96,450.29	183,583.12
Tunisia	63,849.86	51,191.81	81,413.11	118,721.03	83,852.50	168,901.45
Türkiye	379,597.98	319,675.02	443,583.58	698,665.74	503,757.77	964,631.08
United Arab Emirates	13,218.07	10,592.42	16,980.19	79,861.46	54,355.22	114,045.28
Yemen	101,033.70	80,819.19	127,491.49	278,881.22	198,872.11	387,102.82

DALYs = disability-adjusted life-years, MENA = Middle East and North Africa.

**Table 6:** All-ages counts and age-standardized DALYs rates (per 100,000) of anxiety disorder in Middle East and North Africa, stratified by country, 1990 to 2021.

Location	Year					
	1990			2021		
	Value	Lower	Upper	Value	Lower	Upper
Age-standardized DALYs rate (per 100,000)						
MENA	587.75	407.24	806.3	707.08	469.77	994.9
Afghanistan	580.23	391.26	805.45	708.35	439.83	1057.96
Algeria	574.4	387.95	796.03	651.01	400.83	949.52
Bahrain	585.75	387.82	814.46	682.81	412.66	1044.99
Egypt	515.3	348.88	720.58	622.28	363.72	915.86
Iran	801.43	555.47	1093.42	973.7	659.07	1345.76
Iraq	590.37	396.67	818.59	690.9	425.02	1028.28
Jordan	584.19	390.88	828.17	679.18	404.09	1050.26
Kuwait	541.39	360.35	759.7	604.06	377.49	926.51
Lebanon	719.67	483.39	1004.44	981.03	592.83	1442.83
Libya	601.23	399.92	845.71	735.5	441.92	1099.25
Morocco	574.99	388.92	802.42	708.94	442.74	1062.73
Oman	556.33	373.11	778.82	673.09	412.19	1001.77
Palestine	628.28	422.92	887.32	747.83	456.99	1112.11
Qatar	530.36	354.83	742.49	583.07	362.99	829.7
Saudi Arabia	545.46	365.57	769.58	612.79	381.21	918.18
Sudan	576.24	382.42	807.01	681.86	428.05	1046.69
Syrian Arab Republic	615.45	414.74	863.54	759.83	483.21	1145.39
Tunisia	597.96	398.47	840.43	816.84	506.57	1206.50
Türkiye	484.72	343.08	650.68	648.12	385.2	958.6
United Arab Emirates	512.26	350.28	712.03	618.4	374.21	940.32
Yemen	579.24	386.89	805.71	611.69	374.89	903.7
Counts estimates						
MENA	1,908,924.92	1,313,103.31	2,684,061.16	4,508,067.43	2,969,187.28	6,368,320.64
Afghanistan	52,734.77	35,263.86	75,430.97	212,962.59	129,924.95	320,563.99
Algeria	139,381.26	91,893.53	198,019.30	286,953.37	176,141.45	418,472.19
Bahrain	2999.65	1938.83	4311.19	11,201.15	6731.23	16,893.96
Egypt	274,447.46	181,692.46	393,231.36	658,668.85	380,568.80	983,989.28
Iran	423,348.05	292,563.68	590,244.91	864,198.43	587,427.07	1,191,909.69
Iraq	101,355.09	66,849.82	145,981.75	293,838.46	177,147.42	444,955.01
Jordan	20,939.15	13,810.59	30,493.19	88,575.40	52,135.39	137,121.79
Kuwait	9534.59	6202.69	13,672.88	30,496.97	18,803.97	46,382.20
Lebanon	21,005.16	13,989.75	29,732.16	55,916.65	33,923.96	82,474.80
Libya	24,490.23	15,918.49	35,258.81	54,529.73	33,340.45	80,996.37
Morocco	142,402.81	94,743.89	203,755.93	270,396.97	168,855.17	405,942.27
Oman	10,361.78	6699.01	14,784.66	32,703.22	19,846.97	48,697.63
Palestine	11,589.61	7634.42	16,753.58	39,245.20	23,649.70	60,192.27
Qatar	2415.43	1570.85	3477.33	18,559.78	11,154.01	27,034.75
Saudi Arabia	84,001.70	54,257.07	121,400.04	251,808.61	157,586.12	378,771.84
Sudan	107,314.80	70,576.81	154,517.48	300,202.07	185,509.99	468,842.74
Syrian Arab Republic	72,816.45	47,221.24	105,802.40	112,089.77	70,647.01	167,370.54
Tunisia	49,389.03	32,638.57	70,156.55	97,771.02	61,489.29	144,542.28
Türkiye	279,107.75	197,842.38	374,262.38	559,351.19	332,345.44	830,643.25
United Arab Emirates	9588.95	6348.47	13,643.88	61,832.15	37,158.35	96,015.01
Yemen	68,656.93	44,603.72	100,042.37	202,561.17	122,312.39	306,292.06

DALYs = disability-adjusted life-years, MENA = Middle East and North Africa.

## 4. Discussion

Anxiety disorders are among the most common mental health problems in the world, and extensive factors have been stated in the etiology of these disorders. Among these factors are geography, economics, conflicts, and the level of development. This research was conducted to investigate the prevalence of anxiety disorders in MENA, including 21 countries, in the period between 1990 and 2021 based on gender and age disparity.

The first finding obtained from this study showed that the burden of anxiety disorders is higher in MENA compared with the global average. In this region, there were 38 million cases of anxiety disorders, which was equivalent to more than 10% of all cases of anxiety disorders globally. As previous studies have stated, the burden of anxiety disorders has increased year by year, but what is important is that the burden of anxiety disorders varies by region, group, and gender.^[6]^ It has been stated in other studies that in MENA, the burden of mental disorders, especially anxiety, is higher compared with the rest of the world.^[10]^ Different mechanisms may affect the burden of anxiety disorders in MENA, explaining this disparity. Conflicts and wars have been some of the most important challenges in East and North Africa in recent decades, and these are significant reasons for reduced mental health.^[20–23]^ In addition to direct psychological consequences, wars and conflicts are also associated with an increase in the rate of migration and displacement. At the end of 2022, there were 16.2 million internal displacements in this region, which is equivalent to 23% of all internal displacements globally.^[24]^ Of the total displacements in this region, 80% are the result of armed conflict, particularly in Iraq, Libya, Syria, and Yemen.^[24]^ The effects of armed conflict not only directly affect mental health but also impact it by weakening the health system and health infrastructure.^[25]^ Armed conflicts severely impact civilian populations, inflicting both immediate physical harm through violence and enduring mental health challenges, often leaving profound psychiatric scars.^[26]^ This problem has been particularly severe in MENA over recent years and decades, significantly affecting various dimensions of mental health, especially anxiety disorders. Such conflicts exacerbate mental health issues by fostering insecurity, economic hardship, and the collapse of social welfare and healthcare systems. Consequently, this requires heightened focus when analyzing anxiety disorders within this region. Other mechanisms can be mentioned in connection with the burden caused by anxiety disorders in MENA. One issue is the demographic changes that have occurred in recent years. For example, during the past decades, the birth rate increased, and the rate of the young population increased. In this regard, it has been stated that the peak of mental disorders is in adolescence and early adulthood.^[27]^ Extensive research has indicated that women tend to exhibit a greater susceptibility to anxiety disorders compared with men, which is likely influenced by a combination of factors including disparities in socioeconomic conditions, varying levels of exposure to traumatic events, and potential biases in how individuals report mental health challenges. These elements collectively contribute to the observed differences, highlighting the complex interplay of societal influences, psychological experiences, and cultural norms in shaping mental health outcomes across genders.^[28]^

Gender disparity was shown in the prevalence of anxiety disorders; the prevalence in females was more than 1.5 times that in males. This finding is consistent with many existing studies in the research literature.^[29,30]^ A range of biological, psychological, economic, and social mechanisms have been proposed to understand gender differences in anxiety disorders.^[31]^ Therefore, it is necessary to pay attention to these factors in understanding the gender difference. The impact of COVID-19 on the prevalence and burden of anxiety disorders has been significant, with observable trends indicating a marked and concerning rise in such conditions since 2019. The pandemic has not only disrupted daily life on a global scale but has also exacerbated stressors related to health, financial stability, social isolation, and uncertainty about the future. These factors have collectively contributed to a sharp increase in the number of individuals experiencing heightened levels of anxiety. This escalation underscores the profound mental health challenges brought about by the crisis, adding to the already existing pressures faced by people in their personal and professional lives. Some studies specifically examine how COVID-19 influences the regional burden of anxiety.^[14]^

## 5. Conclusion

The trend of the burden of anxiety disorders in global and MENA countries has shown an increasing trend during recent decades that continued since 1990. However, the rate of anxiety disorders in MENA was higher than globally. Changes in the burden of anxiety disorders may also be partly because of the effects of COVID-19 in 2020 to 2021.

## 6. Limitations

This study tried to provide a comprehensive estimate of the burden of anxiety disorders in MENA countries. This research had limitations that are consistent with those of the GBDs. There are problems with the quality and collection of primary data, and inconsistency of data availability. The studied risk factors also have limitations, including the omission of various potentially consequential risk factors and covariates. The effects of COVID-19 are also a challenge. The GBD does not provide a detailed explanation of the precise methodology used to evaluate the impacts of COVID-19. However, an observed increase in anxiety disorders during 2020 and 2021 was likely associated with the pandemic. In some areas, the data may not have been sufficiently clear and accurate, and estimation methods based on the GBD methodology have been used for these areas, so bias in data collection and modeling may exist. More details on the GBD limits are provided elsewhere.^[16,32]^ Other significant limitations include the possibility of diagnostic inconsistencies between the DSM and ICD classification systems, which could influence case identification across different countries. In addition, reporting anxiety disorders as a single, aggregated category may obscure important differences among specific subtypes, such as generalized anxiety disorder, post-traumatic stress disorder, and panic disorder. A limitation of the GBD burden estimates for several countries, particularly Yemen, Libya, and Sudan, is that they rely on limited primary data, resulting in broad uncertainty intervals. In addition, estimates in conflict-affected regions may tend to be conservative. Because of the nature of the data extraction approach, conducting a sensitivity analysis within the manuscript is not feasible. However, a practical sensitivity analysis could offer more valuable insights. For instance, this could involve limiting trend comparisons to countries with higher GBD data quality ratings or assessing whether the post-2019 DALY spike remains consistent after excluding conflict-affected outliers with the largest uncertainty intervals.

The data sources of this study were taken from GBD 2021, which is publicly available.

## Author Contributions

**Conceptualization:** Moien AB Khan, Sohrab Amiri, Abolghasem Yaghoobi.

**Writing – original draft:** Moien AB Khan, Sohrab Amiri, Abolghasem Yaghoobi, Fatemehjan Nikbakht Fini.

**Writing – review & editing:** Moien AB Khan, Sohrab Amiri, Abolghasem Yaghoobi, Fatemehjan Nikbakht Fini.

**Visualization:** Sohrab Amiri.

**Methodology:** Abolghasem Yaghoobi.
